# Pollutants regulate changes in pathological markers of neurodegenerative diseases: a new perspective in environmental toxicology

**DOI:** 10.3389/ftox.2026.1814795

**Published:** 2026-09-03

**Authors:** Xingbo Kou, Qianqian Hu, Yibo Kou, Dinghao An

**Affiliations:** 1 Department of Thoracic Surgery, Shangluo Central Hospital, Shangluo, China; 2 Medical College of Yan’an University, Yan'An, China; 3 Shaanxi University of Chinese Medicine, Xianyang, China; 4 Department of Neurology, Chinese Academy of Medical Sciences and Peking Union Medical College, Beijing, China

**Keywords:** environmental polutants, environmental toxicology, Neurodegenarative diseases, neurology, pathological markers

## Abstract

Neurodegenerative diseases are major global health challenges, with their pathogenesis closely linked to the abnormal aggregation of pathological proteins. Beyond genetic factors, environmental pollutants play key roles in disease onset and progression. Conventional reviews often focus on associations between specific pollutants and particular diseases. This review innovatively concentrates on the common pathogenic mechanisms of core pathological markers (e.g., Aβ, Tau, α-syn, TDP-43), systematically detailing how various pollutants, including air pollutants, metals, and persistent organic pollutants, modulate the generation, aggregation, propagation, and toxicity of pathological proteins, thereby accelerating neurodegenerative processes. We propose an integrated framework of ‘pollutants-pathogenic links-neural network failure,’ bridging environmental toxicology and molecular neurology to offer a unified novel perspective and theoretical foundation for molecular mechanism-based environmental risk assessment and targeted interventions.

## 1 Introduction

Neurodegenerative diseases (NDs), such as Alzheimer’s disease (AD), Parkinson’s disease (PD), and amyotrophic lateral sclerosis (ALS), are leading causes of disability and mortality among the elderly worldwide, imposing a substantial socioeconomic and healthcare burden. Among these, AD alone accounted for approximately 2.0 million deaths and 28.0 million DALYs globally in 2016 (The Lancet, 2019), with global societal costs exceeding USD 1.3 trillion per year (Wimo et al., 2023). As the global population ages, the incidence of NDs continues to rise. However, their etiology remains largely unknown, and effective disease-modifying therapies are scarce, leading to a decline in both quality of life and life expectancy (Karran and De Strooper, 2022). The core pathogenic mechanism of NDs involves misfolding, abnormal aggregation, and intercellular propagation of specific proteins. These pathological markers, such as Aβ, Tau, and α-synuclein (α-syn), are not only the “gold standard” for disease diagnosis but also critical targets for developing disease-modifying therapies, including immunotherapies (de Calignon et al., 2012; Peelaerts et al., 2015).

A critical layer of complexity lies in copathology, where multiple pathological protein aggregates frequently coexist in the brains of individual patients. For instance, over 50% of clinically diagnosed AD patients exhibit mixed pathologies, combining AD hallmarks (Aβ and Tau) with Lewy bodies (α-syn) or TDP-43 pathology (Karanth et al., 2020). This phenomenon is particularly prevalent in the advanced elderly population, contributing to the heterogeneity and mixed nature of clinical symptoms, which poses a significant challenge for precise diagnosis and treatment (Nelson et al., 2019).

Genome-wide association studies indicate that genetic factors only account for a portion of the disease risk, with environmental factors playing a pivotal role as “accelerators” or “modifiers” of disease susceptibility and progression. Pollutants are key environmental modifiers that regulate various neurodegenerative pathologies (Schikowski et al., 2015; Hatcher et al., 2008). Understanding the impact of these environmental factors on pathological markers is fundamental to deciphering disease mechanisms and developing precise diagnostics and targeted therapeutics. In recent years, with the advent of the “exposome” concept, the research paradigm is shifting from studying single toxicants towards a holistic assessment of lifetime environmental exposures (Finch and Kulminski, 2019). While existing reviews have discussed associations between specific pollutants (e.g., PM2.5, pesticides) and NDs (Haddad et al., 2019; Gago-Ferrero et al., 2025), and partial syntheses have covered endocrine-disrupting chemical contributions or emerging pollutant risks (Nabi and Tabassum, 2022; Khan et al., 2026), a systematic review framed around the dynamic disease processes of pathological markers is lacking. This review is the first attempt to integrate current evidence and construct an integrated framework of ‘pollutants-pathogenic links-neural network failure’. It aims to fill this knowledge gap, providing a novel perspective for understanding how environmental factors precisely modulate the molecular progression of NDs and offering a theoretical foundation for developing future preventive strategies against pollution-associated neurodegenerative diseases.

## 2 Pathological markers and disease overview with brief applications

### 2.1 Aβ (Amyloid-beta)

Particularly the aggregation-prone Aβ42, it is produced via proteolytic cleavage of its precursor protein (APP) and aggregates into extracellular amyloid plaques. Aβ pathology is a requisite neuropathological criterion for AD under 2024 NIA-AA/Alzheimer’s Association frameworks and is detected in >95% of neuropathologically confirmed AD cases (Jack et al., 2024; Moscoso and Villain, 2024; Kovacs et al., 2025). However, Aβ deposition is not exclusive to AD. It can also be observed in the brains of cognitively normal elderly individuals, a phenomenon termed “pathological aging” (Lopez et al., 2024). Studies indicate that approximately 60% of cognitively normal individuals over 80 years old have detectable Aβ plaques (Chételat et al., 2013), although their distribution is typically more limited and their burden lower than in AD patients. Furthermore, Aβ pathology is also commonly present in dementia with Lewy bodies (DLB) (45%–50%) and cerebral amyloid angiopathy (CAA) (50%–65%) (Ossenkoppele et al., 2015; Sekiya et al., 2025; Bruce et al., 2025; Charidimou et al., 2022). Importantly, the pathological process of AD is believed to initiate with abnormal Aβ deposition, followed by subsequent changes such as Tau pathology (Jack et al., 2010).

### 2.2 Tau protein

Tau is a microtubule-associated protein. Its hyperphosphorylation leads to loss of function and detachment from microtubules, resulting in intracellular aggregation into neurofibrillary tangles (NFTs) and neuropil threads. Tau is another core pathology of AD, typically emerging after Aβ deposition and showing a stronger correlation with cognitive decline (Braak et al., 2006; Guillozet et al., 2003; Moscoso et al., 2025). In primary tauopathies, Tau pathology is the dominant feature, encompassing approximately 50% of behavioral variant frontotemporal dementia (bvFTD) cases, as well as 100% of progressive supranuclear palsy (PSP) and corticobasal degeneration (CBD) (Neary et al., 1998; Lee et al., 2001). Limited Tau pathology (mainly confined to the entorhinal cortex) can also occur in normal aging brains, but its distribution is distinct from the widespread pathology seen in disease (Bennett et al., 2006).

### 2.3 α-Synuclein

Misfolded α-syn aggregates to form Lewy bodies (LBs) and Lewy neurites, which are definitive markers of synucleinopathies. It is nearly universally present (close to 100%) in cases of PD and DLB, primarily within neurons (Spillantini et al., 1997; McKeith et al., 2017). In multiple system atrophy (MSA) (close to 100%), α-syn mainly aggregates within glial cytoplasmic inclusions (GCIs) in oligodendrocytes (Tu et al., 1998). Notably, approximately 50% of AD patients’ brains also exhibit varying degrees of α-syn pathology (Almeida et al., 2025).

### 2.4 TAR DNA-binding protein 43 (TDP-43)

Under normal conditions, TDP-43 is in the nucleus. In pathological states, it undergoes abnormal phosphorylation, ubiquitination, and mislocalizes from the nucleus to the cytoplasm, where it aggregates. TDP-43 pathology is a primary feature in most ALS cases (>95%) and in approximately 50% of frontotemporal dementia cases (FTD-TDP) (Neumann et al., 2006; Cairns et al., 2007). Additionally, limbic-predominant age-related TDP-43 encephalopathy (LATE) is very common in the advanced elderly, present in about 25%–50% of individuals over 85 years old. It often coexists with AD pathology (50%) and independently exacerbates cognitive impairment (Nelson et al., 2019; Duong and Wolk, 2022; Nelson et al., 2011).

### 2.5 FUS protein

Like TDP-43, its pathological hallmark is loss of nuclear localization and cytoplasmic aggregation. It is primarily found in a minority of ALS (0.3%–0.9%) and FTD cases (5%) (Moens et al., 2025). FUS is a multifunctional protein involved in various aspects of RNA metabolism. Mutations, particularly in the C-terminal domain, can alter the biophysical properties of FUS. It will promote aberrant and irreversible aggregation. Moreover, mutations occur in the nuclear localization signal will lead to impaired nuclear import and subsequent cytoplasmic mislocalization. Once mutations occur in the cytoplasm, the mutant FUS protein is prone to form insoluble aggregates that can disrupt normal RNA processing pathways, contributing to neuronal dysfunction and degeneration. (Chen et al., 2019; Vance et al., 2009). Furthermore, recent studies suggest that FUS aggregation is not merely passive but an active process that can be influenced by environmental factors. Additionally, the interaction of aggregated FUS with other pathological proteins, such as TDP-43, may create a synergistic toxic effect, accelerating disease progression (Moens et al., 2025).

### 2.6 Superoxide dismutase 1 (SOD1)

Gene mutations lead to misfolding and aggregation of the SOD1 protein, observed in approximately 20% of familial ALS cases and 1%–3% of sporadic ALS cases (Rosen et al., 1993; Joyce et al., 2011).

### 2.7 Prion protein (PrP)

The conversion of its normal cellular isoform (PrP^C) into an abnormal, self-replicating pathogenic form (PrP^Sc) is the sole causative agent (100%) of prion diseases, such as Creutzfeldt-Jakob disease (Prusiner, 1991; Legname and Moda, 2017).

### 2.8 Mutant Huntingtin (mHTT)

Produced by an abnormal CAG trinucleotide repeat expansion in the HTT gene, fragments of the mutant protein form aggregates within neurons, serving as the definitive marker (100%) for Huntington’s disease (Huntington’s Disease Collaborative Research Group, 1993; Ross and Tabrizi, 2011).

**FIGURE 1:**
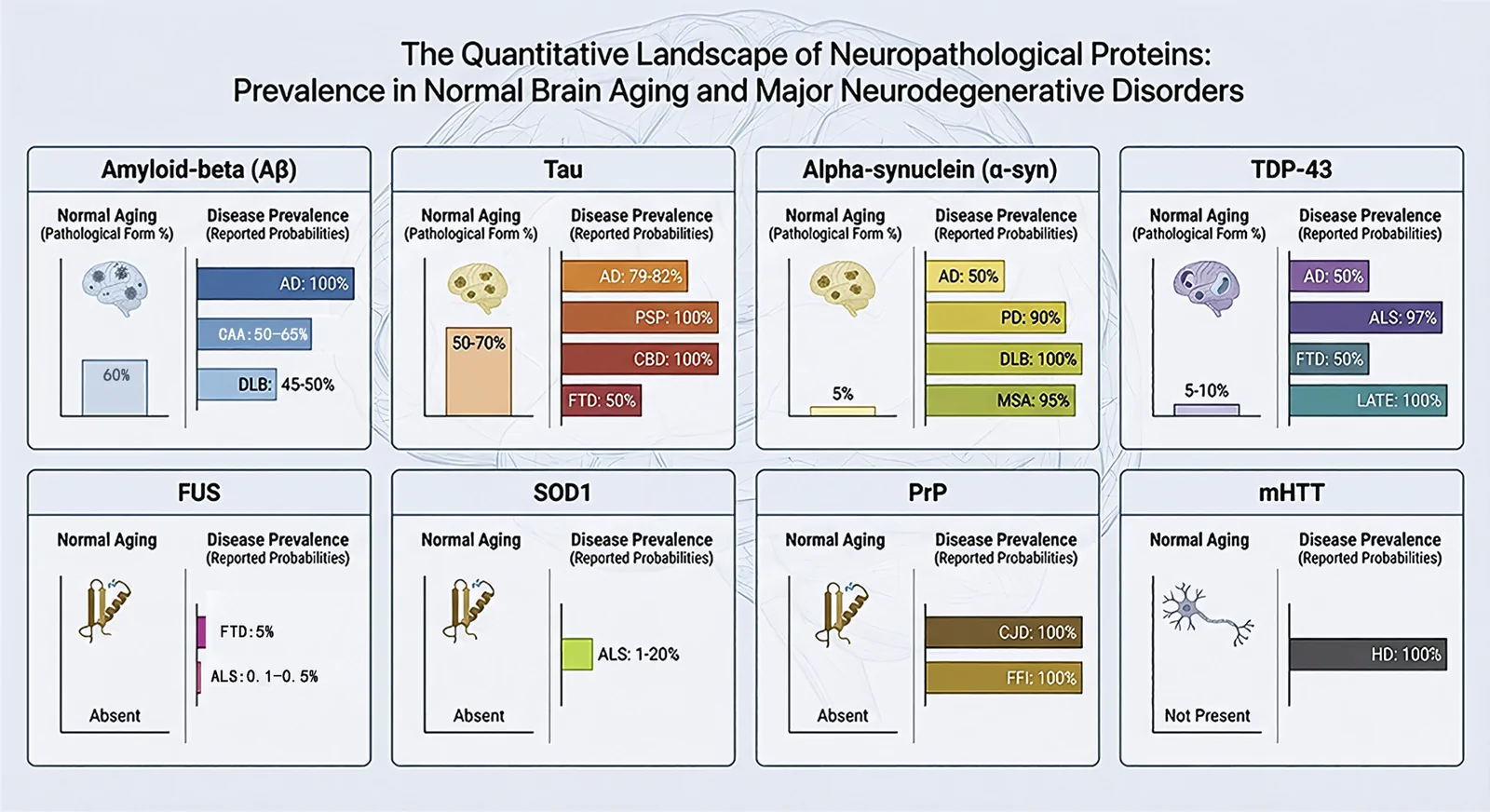
This figure illustrates the spectrum of pathological protein deposition observed in normal brain aging *versus* major neurodegenerative diseases (AD, CAA, DLB, PSP, CBD, FTD, PD, MSA, ALS, HD, CJD, and FFI). For each protein, the left panel indicates the prevalence of pathological forms during normal aging (where “Absent” or “Not Present” signifies no detectable pathology), while the right panel displays the reported probability of protein involvement across different neurodegenerative disorders.

The relationship of common pathogenic proteins with neurodegenerative diseases can be seen in Figure 1. The detection of these pathological markers has evolved from post-mortem/biopsy examinations to the era of *in vivo* biomarkers (Dubois et al., 2014). Techniques such as cerebrospinal fluid (CSF) Aβ42/phosphorylated Tau ratios, plasma P-tau217, Aβ-PET, and Tau-PET imaging have significantly advanced early diagnosis, disease subtyping, and therapeutic evaluation (Hansson et al., 2018). Clearing these pathological proteins or inhibiting their aggregation constitutes a core strategy in current drug development.

## 3 Environmental pollutants as pathological modifiers: classification and overview

### 3.1 Air pollutants

Air pollutants primarily originate from fossil fuel combustion (vehicle exhaust), industrial emissions, power plants, and biomass burning (Daellenbach et al., 2020). Major constituents include fine particulate matter (PM2.5), ultrafine particles (UFPs), black carbon (BC), ozone (O3), nitrogen oxides (NOx), and polycyclic aromatic hydrocarbons (PAHs) adsorbed onto their surfaces (Kim et al., 2013; Dominski et al., 2021; Awasthi et al., 2025; Huang et al., 2024; Oberdörster and Utell, 2002). Inhalation is the primary exposure route. Particulate matter like PM2.5 can be directly translocated to the brain via the olfactory nerve or indirectly facilitate the entry of other neurotoxins by inducing pulmonary/systemic inflammation and increasing blood-brain barrier (BBB) permeability (Galea, 2021; Oberdörster et al., 2004). Substantial epidemiological evidence links air pollution to increased risks of AD and PD, with underlying mechanisms primarily involving the induction of oxidative stress, mitochondrial dysfunction, and systemic neuroinflammation (Teixeira et al., 2024; Chen et al., 2017; Hu et al., 2019), as further supported by recent clinical data linking long-term PM2.5 exposure to accelerated brain atrophy and cognitive decline (Kulcsárová et al., 2025).

### 3.2 Metals/metalloids

Metals and metalloids are ubiquitous in the natural environment and industrial activities. Key examples include lead (Pb) (from old paint, residual leaded gasoline, contaminated water), mercury (Hg) (industrial emissions, consumption of contaminated fish), manganese (Mn) (mining, welding, certain groundwater), arsenic (As) (contaminated groundwater, industrial wastewater), cadmium (Cd) (industrial emissions, smoking, certain foods), and aluminum (Al) (food additives, cookware, antacids) (Yan et al., 2026). Exposure pathways include inhalation (occupational exposure, airborne dust), ingestion (contaminated water and food), and dermal contact (certain industrial products or cosmetics containing metals). They can disrupt brain metal ion homeostasis by mimicking essential metals (e.g., Zn^2+^, Cu^2+^), directly promote Aβ aggregation or Tau phosphorylation, and are potent inducers of oxidative stress (Palma-Lara et al., 2020; Tomljenovic, 2011; Vlasak et al., 2023; Mergler et al., 2007; Wani et al., 2015; Kozlowski et al., 2009).

### 3.3 Persistent organic pollutants (POPs)

Persistent Organic Pollutants encompass pesticides (e.g., historically widely used dieldrin, and paraquat/rotenone highly associated with PD risk), industrial chemicals (e.g., polychlorinated biphenyls [PCBs], brominated flame retardants [BFRs]), and industrial by-products (e.g., dioxins) (Yan et al., 2018; Montano et al., 2022; Zhu et al., 2022; Yu et al., 2016). Due to their high lipophilicity and bioaccumulation potential, dietary ingestion (via contaminated food chains, especially fish, meat, and dairy) is the primary exposure route (Yu et al., 2010). Dermal contact is also significant in certain occupational and accidental exposures. Their association with high neurodegenerative disease risk is particularly notable (Tanner et al., 2011). Their main toxic mechanisms involve disrupting mitochondrial function and energy metabolism and interfering with intracellular signaling (Reddam et al., 2022).

### 3.4 Emerging pollutants

Emerging pollutants are of growing concern and include (The Lancet, 2019): Plastic-related pollutants: nano/microplastics and phthalates used as plasticizers (Wimo et al., 2023). Endocrine-disrupting chemicals (EDCs): such as bisphenol A (BPA) (widely found in plastic containers, food can linings) and per- and polyfluoroalkyl substances (PFAS) (non-stick coatings, waterproof materials) (Karran and De Strooper, 2022). Pharmaceuticals and personal care products (PPCPs): incompletely metabolized drugs, antibiotics, antimicrobials, etc (Kontrick, 2018; Arnesdotter et al., 2025; Evich et al., 2022). Exposure routes for emerging pollutants are diverse, including ingestion (migration from food/water packaging), inhalation (airborne dust), and dermal absorption (cosmetics, personal care products). They can potentially affect neurodevelopment and accelerate neurodegenerative processes through mechanisms like endocrine system disruption, induction of oxidative stress, and epigenetic alterations (Sun and Wang, 2023; Warner and Flaws, 2018).

## 4 Environmental regulation of the toxicity of pathological markers

### 4.1 Induction of abnormal production and aggregation of pathological proteins

This process marks the initial trigger of the entire pathological cascade, encompassing the accumulation of pathological proteins through both overproduction and impaired clearance. The aggregation process progresses from protein monomers to soluble oligomers, then to protofibrils and insoluble fibrils, culminating in large deposits (Hardy and Selkoe, 2002; Ross and Poirier, 2004). Pollutants can initiate this pathology via three primary, often synergistic, mechanisms.

Firstly, the promotion of production. Airborne particulate matter, particularly PM2.5 and ultrafine particles and diesel exhaust particles, serves as a potent source of oxidative stress. In APP/PS1 mice, chronic PM2.5 exposure upregulates β-secretase (BACE1) activity, leading to increased Aβ generation. This is linked to PM2.5-induced oxidative stress, activating related signaling pathways and stimulating reactive astrocytes and microglia to release pro-deposition factors (Kilian et al., 2023; Fonken et al., 2011). Certain metals (e.g., Al^3+^) can mimic signaling molecules to modulate the activity of APP processing enzymes like BACE1, increasing Aβ production (Praticò et al., 2002). Polycyclic aromatic hydrocarbons (PAHs) adsorbed onto PM2.5 surfaces can also promote the generation of pathological markers (Olasehinde and Olaniran, 2022). Early life exposure to bisphenol A (BPA) may epigenetically regulate the expression of APP processing enzymes, promoting amyloidogenesis (Somm et al., 2009; Engin and Engin, 2021). Pesticides like organophosphates can lead to synaptic acetylcholine accumulation, indirectly activating APP processing pathways (Chen et al., 2024). The herbicide paraquat can upregulate α-synuclein gene transcription, increasing its production (Manning-Bog et al., 2002).

Secondly, direct promotion of aggregation. Beyond increasing production, oxidative environments cause post-translational modifications of multiple pathological proteins. For instance, reactive oxygen and nitrogen species can induce nitration and hyperphosphorylation of α-synuclein and tau, significantly enhancing their propensity to form insoluble oligomers and fibrils (Abasi et al., 2024; Horiguchi et al., 2003). Diesel exhaust particles (DEPs) can directly act on neurons, inducing abnormal phosphorylation and oligomerization of α-syn. Chronic ozone exposure activates tau kinases like GSK-3β, resulting in tau hyperphosphorylation and neurofibrillary tangle formation (Velázquez-Pérez et al., 2021; Hartz et al., 2008). The direct interaction between metal ions and pathological proteins represents a particularly well-characterized toxic axis. Divalent and trivalent metal ions, including aluminum, copper, iron, zinc, and manganese, can directly bind specific domains of proteins such as Aβ, α-syn, tau, TDP-43, and PrP (Gerber et al., 2017; Moons et al., 2020). While involved in Aβ metabolism under physiological conditions, their homeostatic imbalance can accelerate the rapid aggregation of pathological proteins. Aluminum has long been implicated in neurodegeneration, promoting tau hyperphosphorylation and stabilizing Aβ aggregates (Praticò et al., 2002; Mold et al., 2021). Copper and iron participate in redox cycling, generating highly reactive hydroxyl radicals via the Fenton reaction, leading to oxidative cross-linking of Aβ and α-syn into stable, toxic oligomers (Kozlowski et al., 2009; Moons et al., 2020; Lane et al., 2018). Metals like zinc and copper also disrupt the physiological liquid-liquid phase separation (LLPS) of RNA-binding proteins like FUS and TDP-43, shifting the LLPS equilibrium towards pathological solid-state aggregation (Li F. et al., 2022; Preethi et al., 2021). Arsenic exposure induces cytoplasmic TDP-43 aggregation; *in vitro* studies show arsenite treatment induces cellular stress, leading to aberrant TDP-43 phosphorylation and mislocalization from the nucleus to the cytoplasm, forming insoluble aggregates (Gasset-Rosa et al., 2019; Foster et al., 2021). In animal models of manganese exposure, manganese has been found enriched in Lewy bodies and co-localized with α-syn (Verina et al., 2013), and it can also drive cytoplasmic mislocalization and aggregation of TDP-43, stabilizing its misfolded conformation (Foster et al., 2021; Hester et al., 2022). Polychlorinated biphenyl (PCB) congeners may promote tau hyperphosphorylation through multiple kinase pathways. In particular, PCB 37 exerts divergent effects on neuronal development, like apoptosis and dendritic arborization, partly via aberrant cleavage of cytoskeletal proteins such as tau (Badley et al., 2025). This is consistent with earlier classic syntheses establishing PCBs and pesticides as high-priority neurotoxic modifiers of protein aggregation (Chin-Chan et al., 2015). Paraquat can accelerate the *in vitro* rate of α-synuclein fibril formation markedly (Manning-Bog et al., 2002). Some pollutants may also impair the function of molecular chaperones like heat shock proteins, weakening their capacity for proper nascent peptide folding and refolding of misfolded proteins, thereby promoting the aggregation of all pathological proteins (Cicchetti et al., 2009). Emerging pollutants like engineered nanomaterials may exacerbate the liquid-to-solid phase transition of pathological proteins by providing heterogeneous surfaces that act as nucleation catalysts (Linse et al., 2007; John et al., 2018; Yang et al., 2025). Furthermore, industrial chemicals such as formaldehyde are potent carbonyl stressors, directly forming advanced glycation end products (AGEs) with lysine residues on tau and Aβ, causing aberrant cross-linking and stabilizing pathological aggregates (Lu et al., 2013; Kothandan et al., 2024).

Thirdly, inhibition of clearance. Multiple pollutants can impair the ubiquitin-proteasome system (UPS) and the autophagy-lysosomal pathway. Functional decline of these two major cellular clearance systems is a common early event in many NDs (Radad et al., 2006). PM2.5 may hinder Aβ clearance by inducing insulin resistance (Huang et al., 2026). Animal models of developmental lead exposure show significantly elevated brain tau phosphorylation in adulthood; a mechanism potentially related to lead’s persistent disruption of calcium signaling and the balance of protein phosphatase/kinase systems (Dash et al., 2016; Cory-Slechta et al., 2008). Pesticides like rotenone and paraquat are classic mitochondrial toxins, inducing an energy crisis and severe oxidative stress. This not only promotes the aggregation of proteins like α-syn and TDP-43 but also inhibits the UPS and autophagy, weakening the cell’s ability to clear misfolded proteins such as mHTT or SOD1 (Radad et al., 2006; Nisticò et al., 2011). PCBs can also inhibit proteasome activity, leading to the accumulation of misfolded proteins (Devi et al., 2022; Johnston and Samant, 2021). The multifaceted, synergistic assault by diverse environmental pollutants lays the foundational molecular pathological basis for protein misfolding and aggregation.

### 4.2 Enhancing the intercellular transmission of pathological proteins

The transcellular spread of pathological proteins is a crucial mechanism for disease progression and propagation within the brain (Hardy and Selkoe, 2002). This transmission follows specific neuroanatomical connections, reaching different and broader brain regions along distinct pathways (Liu et al., 2012). This process is significantly exacerbated by environmental pollutants. Pollutants can disrupt plasma membrane integrity, increase its permeability, and facilitate the release of pathological oligomers. More importantly, they can perturb extracellular vesicle biology, for instance, by stimulating the generation and release of exosomes, which are important vectors for the intercellular transmission of pathological proteins (Agyare et al., 2013). Furthermore, pollutant-induced neuroinflammation alters the extracellular and neuroimmune microenvironment, affecting the release, stability, and uptake of pathological proteins (Devi et al., 2022).

In this context, core neuroinflammatory executors driven by pollutant exposure—microglia and astrocytes—are pivotal. Air pollutants like diesel exhaust particles and PM2.5 induce a chronic pro-inflammatory state. This chronic pro-inflammatory state not only damages neurons but also prompts microglia to adopt a phagocytic phenotype that is inefficient at degrading pathological proteins like Aβ, tau, α-synuclein, and TDP-43 (Awasthi et al., 2025; Hu et al., 2019; Yan et al., 2026; Moulton et al., 2012; Thei et al., 2018; Scheiblich et al., 2021; Zelic et al., 2025). Additionally, particulate matter that reaches the brain parenchyma and systemic inflammation triggered by pollutants can compromise the blood-brain barrier, causing endothelial activation and tight junction disruption. This not only allows peripheral inflammatory mediators into the brain but also alters the brain’s extracellular environment, increasing neuronal secretion of exosomes and other extracellular vesicles carrying pathogenic agents like Aβ, tau, or α-syn, thereby promoting the diffusion of extracellular pathological oligomers (Sweeney et al., 2018; Yun et al., 2019). Engineered nanomaterials and UFPs can acquire a protein corona or be surface functionalized to bind pathological proteins. The particle-protein complexes may then translocate across biological barriers via endocytosis or transcytosis (Han et al., 2023; Liu et al., 2023; Kim et al., 2012). Metal ions and lipophilic POPs may integrate into cell membranes, increasing permeability and making intracellular pathological oligomers more likely to be expelled into the extracellular space. Stress conditions induced by pesticides or heavy metals have been shown to alter the composition, size, and abundance of exosomes released by neurons and glia. Under such stress, cells may preferentially package misfolded proteins like TDP-43, FUS, or mutant SOD1 into exosomes as a default clearance mechanism, significantly increasing the pathological protein content in neuron-derived exosomes and enhancing the ability of neighboring neurons to uptake these exosomes, thus accelerating pathological propagation (Bátiz et al., 2015; Saman et al., 2012; Gupta and Pulliam, 2014; Bernal-Vicente et al., 2026; Darabi et al., 2024). Certain dioxin-like PCB congeners can upregulate extracellular vesicle/exosome release from glial and immune cells via inflammatory pathways (Wang et al., 2019). Independently, EVs are established vehicles for the prion-like intercellular transmission of Tau and α-synuclein in neurodegenerative contexts (Ruan, 2022; Melachroinou et al., 2024). Thus, PCB-elevated EV biogenesis may amplify pathological protein spread, although this link remains to be directly demonstrated in PCB models. Besides, a recent work confirms that ultrafine particle exposure exacerbates α-syn propagation specifically via exosomal routes (Lee et al., 2023). The organophosphate pesticide chlorpyrifos, through its neurotoxic effects, can impair synaptic function, potentially increasing the release of pathological proteins into the synaptic cleft (Moyano et al., 2019). PCBs and some endocrine disruptors can further alter the lipid composition of these vesicles, potentially increasing their stability in the extracellular space and their fusion efficiency with target cell membranes. Additionally, the classic transmissible agent, the prion protein, has been found associated with exosomes (Gupta and Pulliam, 2014). Although direct evidence linking pollutants to increased exosomal release of prions is limited, the profound activation of cellular stress and trafficking pathways by industrial chemicals provides a plausible mechanistic link for enhanced release of infectious units (Hartmann et al., 2017). Various pollutants induce neuroinflammation, altering the transmission context. Activated microglia, while attempting to phagocytose pathological proteins, may fail to degrade them effectively, instead carrying the proteins to more distant brain regions. Pollutants inhibiting autophagy can lead to the accumulation of autophagic vesicles and potential non-classical secretion, which may contain undegraded pathological proteins. If apoptotic or necrotic pathways are activated, they directly release cellular contents, including aggregated pathological proteins, into the extracellular environment for phagocytosis by neighboring cells (Johnston and Samant, 2021; Perea et al., 2018; Lee and Ye, 2022). Pollutants like perfluoroalkyl substances directly attacking the BBB may increase passive leakage of intracellular oligomers like Aβ or tau, while also making cells more susceptible to uptake of extracellular aggregates through compromised membrane domains (Wang et al., 2011; Senatorov et al., 2019). In human studies, Tau-PET imaging has vividly demonstrated the propagation pattern of Tau pathology along specific neuroanatomical connections (from the entorhinal cortex to hippocampus to association cortices) (Vanderlinden et al., 2025; Groot et al., 2022). In summary, environmental pollutants likely act as “boosters,” accelerating the rate of pathological transmission by fostering a fierce inflammatory environment, activating non-canonical secretory pathways, disrupting intercellular communication systems, and compromising biological barriers.

### 4.3 Exacerbating pathological protein-mediated cytotoxicity

This mechanism pertains to the direct actions of pathological proteins, including disruption of cell membranes and organelles, impairment of synaptic function, induction of oxidative stress, interference with intracellular trafficking, promotion of neuronal death, and clogging of cellular “clearance systems.” (Ballatore et al., 2007) Pollutants and pathological proteins can exhibit various synergistic effects, amplifying toxicity and dynamic pathological processes.

Many pathological proteins, such as α-syn, Aβ, and mutant SOD1, directly or indirectly impair mitochondrial complex function, homeostasis, and mitochondrial dynamics (Spillantini et al., 1997; Joyce et al., 2011; Saman et al., 2012). Pollutants deliver an additional attack. PM2.5 bursts induce mitochondrial membrane potential collapse and ROS bursts; when combined with the effects of Aβ oligomers or α-synuclein, the damage to mitochondria is additive or synergistic, leading to severe ATP production deficits and depletion (Moulton and Yang, 2012; Thei et al., 2018). Manganese accumulates in mitochondria, inhibiting complex I, while α-syn oligomers also impair mitochondrial function. It also impairs glutamate uptake, prolonging its synaptic presence and thereby amplifying excitotoxicity—a mechanism particularly detrimental in TDP-43- or SOD1-related ALS, where motor neurons are extremely vulnerable. Their combined action in substantia nigra dopaminergic neurons—inherently vulnerable due to high oxidative stress and weak calcium buffering capacity—leads to selective vulnerability (Zelic et al., 2025; Sidoryk-Wegrzy et al., 2013; Harischandra et al., 2019). Metals (e.g., iron, copper) bound to Aβ or α-synuclein can catalyze site-specific ROS generation via the Fenton reaction. Lead, combined with Tau pathology, not only impairs presynaptic neurotransmitter release but also synergizes with postsynaptic dysfunction, severely damaging long-term potentiation (LTP) and affecting learning and memory (Wani et al., 2015; Hester et al., 2022; Bátiz et al., 2015). Rotenone and paraquat are potent mitochondrial toxins; their inhibition of complex I generates massive ROS, forming a positive feedback loop with ROS produced by α-syn itself, overwhelming cellular antioxidant defenses and causing extensive damage to lipids, proteins, and DNA (Tanner et al., 2011; Betarbet et al., 2000). Additives like paraben preservatives and plasticizers (e.g., BPA, DEHP) can disrupt electron transport and induce mitochondrial permeability transition (Dehghani et al., 2023; Martínez-Razo et al., 2025). Pathological proteins like Aβ oligomers and PrP^Sc can form pore-like structures or overactivate glutamate receptors (e.g., NMDA receptors), working in concert to exacerbate calcium overload and neuronal death (Mercer and Harris, 2023; Taniguchi et al., 2022). Air pollution-derived nanoparticles and certain industrial solvents can directly modulate ion channel function or lipid membrane fluidity, disrupting delicate electrochemical gradients (Moulton and Yang, 2012). Furthermore, oxidative stress induced by metals and pesticides damages endogenous calcium buffering systems in the endoplasmic reticulum and mitochondria, weakening the cell’s capacity to sequester calcium surges triggered by pathological proteins. This synergistic failure transforms a regulated signal into a persistent cytotoxic trigger, activating calpains and other proteases that dismantle the neuronal cytoskeleton (Ermak and Davies, 2002). Certain PCB congeners can cause endoplasmic reticulum calcium store release, leading to aberrant increases in intracellular calcium concentration (McCann et al., 2021). This synergizes with calcium dyshomeostasis caused by various pathological protein oligomers to induce apoptosis (Hyeon et al., 2012). Dieldrin can inhibit the expression of key autophagy proteins, thereby blocking the cell’s ability to clear misfolded proteins, allowing pathological protein aggregates to persist and their toxicity to intensify (Sun et al., 2005; Gezer et al., 2020). As mentioned, PCBs and some metals directly inhibit the activity of the 26S proteasome, a common mechanism leading to the accumulation of various aggregation-prone proteins, including mHTT and mutant SOD1 (Montano et al., 2022; Hester et al., 2022; McCann et al., 2021; Ribas-Fitó et al., 2001). Pollutants can broadly inhibit cellular autophagy by affecting autophagosome formation, fusion with lysosomes, or lysosomal acidification and function, impacting the clearance of proteins like Aβ, α-syn, and TDP-43 (Rahman et al., 2023). In Alzheimer’s disease models, BPA exposure counteracts the protective effects of estrogen (which normally suppresses Aβ production and promotes its clearance), thereby unleashing the full cytotoxic potential of Aβ plaques and oligomers (Johnston and Samant, 2021; Moyano et al., 2019). Similarly, in Parkinson’s or tauopathy models, the loss of hormone-mediated protection renders neurons more susceptible to α-syn or phosphorylated tau-induced synaptic stripping and metabolic stress (Johnston and Samant, 2021; Senatorov et al., 2019). Environmental pollutants act as potent force multipliers, exacerbating pathological protein-mediated cytotoxicity by impacting energy production, calcium dynamics, clearance systems, and signaling pathways.

### 4.4 Initiating and amplifying the vicious cycle of neuroinflammation

This process acts as a “booster” and amplifier of disease progression and is a central part of pollutant action. Neuronal damage and the release of pathological proteins continuously activate the brain’s immune cells—microglia and astrocytes—inducing inflammation. This chronic, low-grade inflammatory environment further damages neurons, persistently promoting the production and aggregation of pathological proteins (Heneka et al., 2025). Spatiotemporal transcriptomic studies confirm that glial cells play a protective role early on but transition to a damaging, pro-inflammatory phenotype later (Masuda et al., 2019). Pollutant-induced epigenetic reprogramming of microglia can accelerate phenotypic switch (Kalenik et al., 2025). Moreover, pollutants can disrupt this temporal sequence early on, driving glial phenotypic switching and the release of large quantities of inflammatory cytokines such as IL-1β and TNF-α. These inflammatory factors compromise BBB integrity, allowing more peripheral immune cells and toxins to enter the brain, creating a self-reinforcing vicious cycle. They also inhibit the phagocytic function of glial cells, transforming them from scavengers to destroyers (Fonken et al., 2011; Marín-Palma et al., 2023; Li et al., 2025).

Airborne particulate matter is not merely an inert irritant. Pathogen-associated molecular patterns (PAMPs, e.g., endotoxin LPS) adsorbed onto its surface can be recognized by Toll-like receptor 4 (TLR4) on microglia, directly polarizing them from a resting M2 state to a pro-inflammatory M1 state, releasing copious IL-1β and TNF-α (Islam et al., 2025; Skrzypczak-Wiercioch and Sałat, 2022). Ultrafine particles can be phagocytosed by microglia, leading to lysosomal damage and ROS production, which in turn activates the NLRP3 inflammasome. This results in caspase-1-mediated maturation and release of potent pro-inflammatory cytokines like IL-1β and IL-18, which can upregulate BACE1 expression in neurons, promoting further Aβ generation. Inflammatory cytokines can also enhance the binding and internalization of these vesicle-associated or free protein aggregates (Hou et al., 2022; Heneka et al., 2018). Heavy metals (lead, mercury, cadmium, arsenic) trigger a vicious cycle of oxidative stress and inflammation (NLRP3 inflammasome, NF-κB) by inducing excessive production of ROS and reactive nitrogen species (RNS) (Hester et al., 2022; Park and Youn, 2013; Fan et al., 2023). TCDD (dioxin), a high-affinity ligand for the aryl hydrocarbon receptor (AhR), activates astrocytes upon binding, causing them to release pro-inflammatory factors and neurotoxic substances (Sha et al., 2025). Industrial chemicals like nanoplastics and PCBs act on the same receptors, thereby lowering the threshold for subsequent inflammatory bursts (Yu et al., 2025).

Under chronic inflammation, microglial capacity to phagocytose pathological proteins and dead cell debris declines, and they shift to a damaging phenotype, exacerbating neuronal death in the presence of α-synuclein, Aβ, SOD1, or TDP-43 aggregates. Inflammatory mediators like nitric oxide (NO) and prostaglandins are directly toxic to neurons, releasing more damage-associated molecular patterns (DAMPs) that further activate microglia (Gao et al., 2023; Calabrese et al., 2007). Inflammatory cytokines can disrupt the tight junctions of the BBB, allowing peripheral immune cells and more toxins to infiltrate the brain. IL-1α, TNFα, and C1q released by activated microglia can induce A1-type astrocytes. These astrocytes lose their normal synaptic support and homeostatic trophic functions and secrete neurotoxic substances, accelerating neuronal death. Activated microglia and astrocytes continue to release large amounts of ROS, RNS, pro-inflammatory cytokines, and excitotoxic levels of glutamate, directly damaging neurons (Galea, 2021; Liddelow et al., 2017). This damage, in turn, leads to further release and modification of pathological proteins, which act as new signals stimulating stronger glial activation.

Chronic pollutant exposure may reprogram neuroglial cells via epigenetic mechanisms (e.g., DNA methylation, histone modifications), placing them in a state that mounts a more intense and prolonged inflammatory response to subsequent stimuli (Yeh and Ikezu, 2019; Wang Z. et al., 2022). Beyond central effects, pollutants also exert peripheral influence. Many pollutants, such as PFAS, heavy metals like lead, and microplastics, induce low-grade chronic inflammation in peripheral organs like the liver, gut, and lungs. They disrupt intestinal barrier integrity, leading to bacterial LPS leakage and promoting the release of circulating pro-inflammatory cytokines (Li et al., 2025; Ma et al., 2026; Li et al., 2024; Wang Q. et al., 2022). These persistent peripheral inflammatory signals communicate with the central nervous system via multiple routes: through compromised areas of the BBB, active transport, or by stimulating vagal afferent fibers. This communication primes or sensitizes glial cell populations. For instance, in tauopathy or Huntington’s disease models, peripheral inflammatory stimuli from pollutant exposure can lead to an exacerbated cascade triggered by subsequent pathological protein accumulation (Ising and Heneka, 2023; Blusch and Björkqvist, 2025; Hoogland et al., 2015).

### 4.5 Neural network disintegration and systemic failure

This is the ultimate outcome of the pathological cascade. As extensive neuronal death, synaptic loss, and glial dysfunction accumulate, the integrated effect is severe synaptic depletion, neuronal loss, and brain atrophy in specific regions (e.g., entorhinal cortex and hippocampus in AD, substantia nigra in PD, putamen and pons in MSA) (Pini et al., 2016; Su et al., 2025). Macroscopically, this manifests as ventricular enlargement and widened sulci. Local neural circuits and large-scale functional networks, such as the default mode network and the basal ganglia motor circuit, progressively disintegrate. When the functional compensation of neural networks is exhausted, irreversible, progressively worsening cognitive impairment, motor symptoms, or neuropsychiatric abnormalities emerge clinically (Andrews-Hanna et al., 2007; Wang et al., 2024).

Pollutants and pathological proteins act synergistically to directly damage synaptic structure and function. Combined direct toxic injury and inflammatory pruning lead to loss of synaptic density, abnormal neural communication, and disruption of neural connections (Wu et al., 2020; Schafer et al., 2012). Organophosphate pesticides cause acute toxicity by inhibiting acetylcholinesterase, leading to excitotoxic damage at cholinergic synapses, while long-term exposure downregulates the expression of key synaptic proteins like synaptophysin and PSD-95 (Chen et al., 2024; Zhou et al., 2023). Heavy metals intensify this assault by stabilizing Aβ and α-synuclein into soluble oligomeric forms, which are particularly toxic to synapses. These metal-coordinated, toxicity-enhanced oligomers embed themselves into synaptic membranes, disrupting glutamate receptor trafficking (e.g., NMDA and AMPA receptors) and causing dendritic spine atrophy (Johnston and Samant, 2021; Thibaudeau et al., 2018). Beyond the synapse, the vital axonal transport system of neurons teeters on the brink of collapse. Pathological tau, hyperphosphorylated due to pollutant-exacerbated kinase activation, detaches from microtubules, destabilizing transport (Ballatore et al., 2007). Industrial solvents and their metabolites, such as acrylamide and n-hexane metabolites (2,5-hexanedione), directly form covalent adducts with neurofilament and tubulin proteins, crosslinking the cytoskeleton and causing severe transport deficits—a core pathology of axonopathy and an early feature in tauopathies and ALS (LoPachin et al., 2003; Gold, 1987). Furthermore, mitochondrial toxins like rotenone deprive axonal transport motor proteins (kinesin and dynein) of the ATP required for function, causing obstruction and the accumulation of dysfunctional organelles like damaged mitochondria and autophagic vesicles within swollen axons, promoting neuronal apoptosis (Rubio et al., 2025; Millecamps and Julien, 2013).

Population imaging studies have found that older adults living long-term in areas with high PM2.5 pollution show significantly reduced intrinsic connectivity within the default mode network (DMN) on resting-state fMRI, correlating with higher cerebral Aβ burden (Lucht et al., 2022; Glaubitz et al., 2022). Individuals with developmental lead exposure exhibit abnormal connectivity in the frontoparietal network responsible for executive function in adulthood, potentially related to synergistic effects between lead and Tau pathology (Lee et al., 2022). Neurotoxins like MPTP and the pesticide paraquat specifically damage substantia nigra dopaminergic neurons, causing dysfunction in the basal ganglia-thalamocortical motor circuit, directly leading to PD motor symptoms (Langston et al., 1983; Sharma and Mittal, 2024). Aluminum has shown toxicity to hippocampal neurons in animal experiments, impairing spatial learning and memory, acting synergistically with the disruption of the medial temporal lobe memory circuit and functional connectivity by Aβ and Tau pathology (Kandimalla et al., 2016).

Moreover, many pollutants like air pollution and heavy metals are not selective neurotoxins; they concurrently cause multi-organ damage that reduces cardiac output and blood oxygen saturation, directly limiting cerebral perfusion (Yan et al., 2026; Bo et al., 2017). The resulting chronic cerebral hypoperfusion is a known driver of white matter lesions, blood-brain barrier disruption, and increased susceptibility/reduced resilience to the toxicity of various pathological proteins (Kitaguchi et al., 2009; Gurol, 2013). Many endocrine-disrupting chemicals and persistent organic pollutants are recognized obesogens and diabetogens, promoting peripheral insulin resistance, dyslipidemia, and vascular dysfunction, which translates directly into central insulin resistance in the brain (Kim et al., 2019; Li L. et al., 2022; Fang et al., 2015). Since insulin signaling in neurons is crucial for synaptic plasticity, glucose uptake, and inhibition of GSK-3β (a key tau kinase), its impairment by pollutants like BPA creates a brain environment that is both energy-deficient and prone to tau and Aβ pathology (Agyare et al., 2013; Flores et al., 2022; Kellar and Craft, 2020). Environmental pollutants exert sustained pressure on neural networks, internally eroding synaptic connections and cellular metabolism. The irreversible collapse of neural circuits and comprehensive biological failure ultimately manifests as the systemic failure of memory and cognitive functions, propelling neurodegenerative diseases into their terminal stages.

**FIGURE 2:**
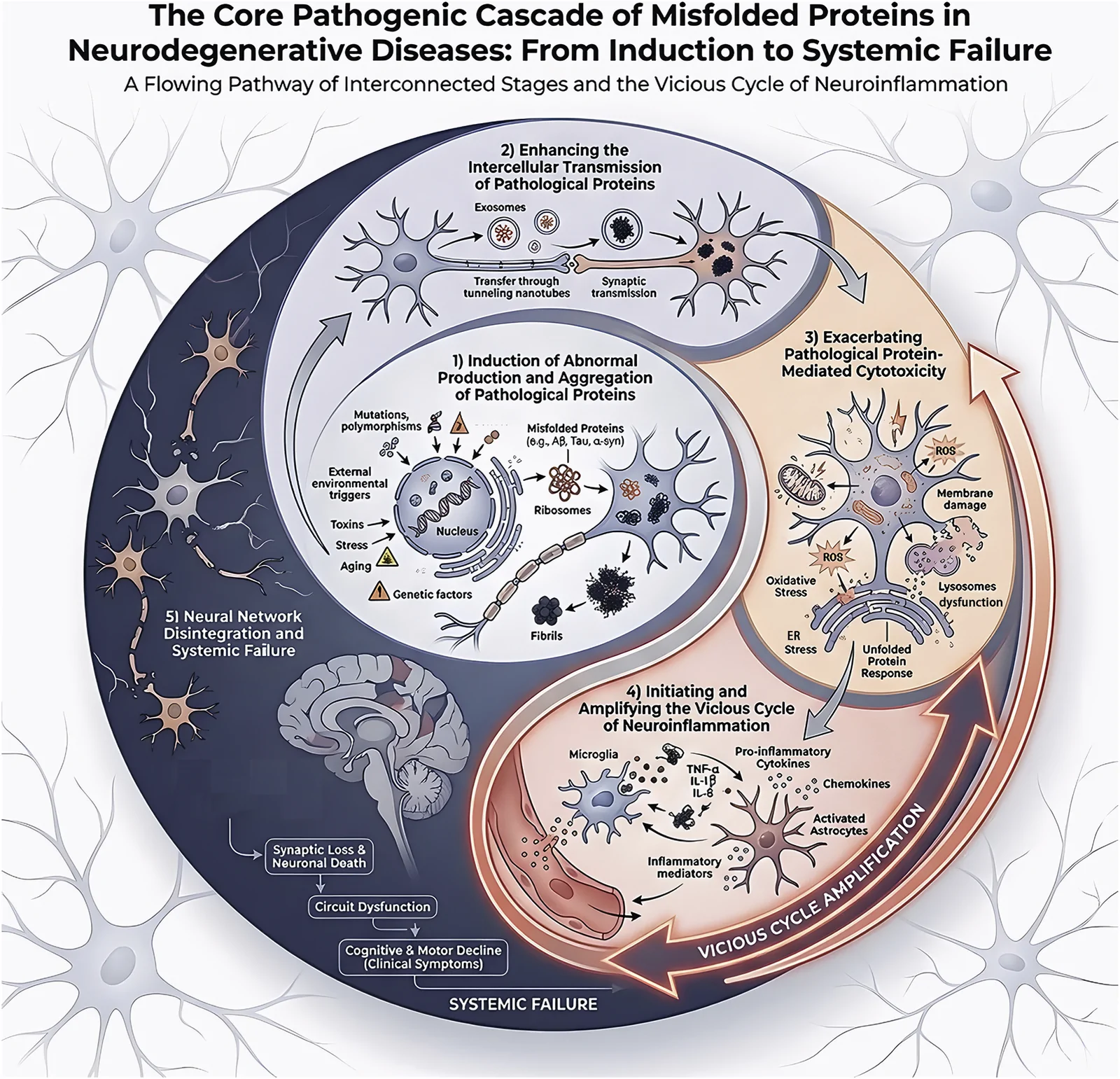
The Core Pathogenic Cascade of Misfolded Proteins in Neurodegenerative Diseases: From Induction to Systemic Failure. This circular schematic depicts the interconnected stages of the neurodegenerative process, culminating in systemic failure. Stage 1: Induction of abnormal production and aggregation of pathological proteins, triggered by genetic mutations, epigenetic modifications, environmental stressors, toxins, and aging. Stage 2: Enhanced intercellular transmission via exosomes, tunneling nanotubes, and synaptic transfer. Stage 3: Exacerbation of cytotoxicity through endoplasmic reticulum (ER) stress, mitochondrial dysfunction, oxidative stress, lysosomal impairment, and unfolded protein response activation. Stage 4: Initiation and amplification of neuroinflammation via microglial and astrocytic activation, releasing pro-inflammatory cytokines (TNF-α, IL-1β, IL-8) and chemokines, creating a vicious cycle. Stage 5: Neural network disintegration and systemic failure, resulting in synaptic loss, neuronal death, circuit dysfunction, and subsequent cognitive and motor decline.

**FIGURE 3:**
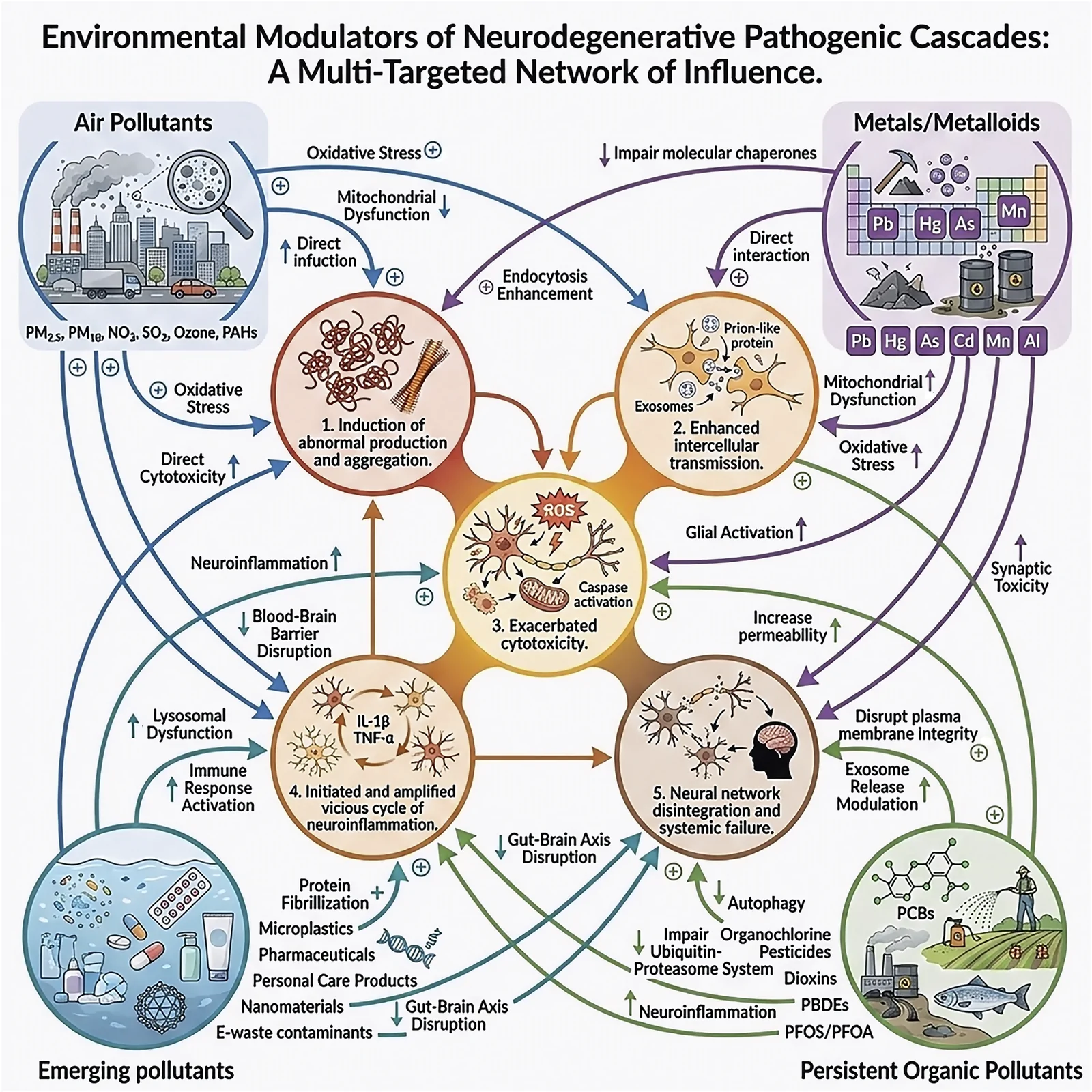
Environmental Modulators of Neurodegenerative Pathogenic Cascades: A Multi-Targeted Network of Influence. This network diagram illustrates how environmental pollutants disrupt neurodegenerative pathogenic cascades in many ways. Four primary routes of exposure include air pollutants (PM2.5, PM10, NO_x_, SO2, Ozone, PAHs), metals/metalloids (Pb, Hg, As, Cd, Mn, Al), persistent organic pollutants (PCBs, dioxins, PBDEs, PFOS/PFOA, organochlorine pesticides) and emerging pollutants (microplastics, pharmaceuticals, personal care products, nanomaterials, e-waste contaminants). These environmental factors converge on five critical stages of neurodegeneration. Arrows indicate regulatory interactions. An upward arrow indicates a facilitating process, while a downward arrow indicates functional impairment or decline. A plus sign represents a facilitating process.

The common mechanisms of pathological markers can be seen in Figure 2, and the mechanisms by which environmental pollutants affect and regulate pathological markers can be seen in Figure 3. As summarized in Table 1, which can be found in supplementary materials, various environmental pollutants contribute to neurodegeneration.

**TABLE 1:** Summary of major environmental pollutants, their sources/exposure routes, and their impacts on neurodegenerative outcomes.

Pollutant	Sources and exposure routes	Key mechanisms (acute and chronic)	Corresponding specific neurodegenerative outcomes	References
Air pollutants	Sources: Vehicle exhaust, industrial emissions, biomass burning Exposure: Inhalation of PM2.5, NOx, SO2, O3, and PAHs	Acute: Direct cytotoxicity, oxidative stress, mitochondrial dysfunction, and blood-brain barrier disruption Chronic: Sustained neuroinflammation, accumulation of advanced glycation end-products, and activation of microglia	Aβ, tau α-syn	Kilian et al. (2023); Fonken et al. (2011); Abasi et al. (2024); Horiguchi et al. (2023); Velázquez-Pérez et al. 2021; Hartz et al. (2008); Heneka et al. (2018)
Metals/Metalloids	Sources:​Mining, smelting, contaminated water, industrial waste Exposure: Ingestion, inhalation of dust	Acute: Direct binding to proteins, generating ROS, and enzyme inactivation Chronic: Impaired molecular chaperones, disruption of kinase/phosphatase balance, and lysosomal dysfunction	Aβ, α-syn Tau, TDP-43, PrP	Praticò et al. (2002); Gerber et al. (2017); Moons et al. (2020); Mold et al. (2021); Lane et al. (2018); Gasset-Rosa et al. (2019); Foster et al. (2021); Verina et al. (2013); Hester et al. (2022)
Persistent organic pollutants	Sources: Pesticides (dieldrin, rotenone, paraquat), industrial chemicals (PCBs, dioxins) Exposure: Dietary intake, occupational contact	Acute: Mitochondrial complex I inhibition, direct disruption of synaptic transmission Chronic: Proteasome/autophagy blockade, epigenetic alterations (e.g., DNA methylation), AhR receptor activation	Tau, α-syn TDP-43, mHTT, SOD	Montano et al. (2022); Tanner et al. (2011); Hester et al. (2022); Radad et al. (2006); Nisticò et al. (2011); Devi et al. (2022); Moyano et al. (2019); Dehghani et al. (2023); Hyeon et al. (2012); Gezer et al. (2020); Rahman et al. (2023); Yu et al. (2025)
Emerging pollutants	Sources: Plastic degradation, consumer products (cosmetics, packaging), BPA, phthalates, PFAS, nanoplastics Exposure: Contaminated food/water, dermal contact	Acute: Endocrine disruption, epigenetic regulation (histone modification) Chronic: ER stress, unfolded protein response dysregulation	Aβ, tau α-syn	Kontrick (2018); Somm et al. (2009); Linse et al. (2007); John et al. (2018); Yang et al. (2025); Liu et al. (2023); Martínez-Razo et al. (2025); Mercer and Harris (2023); Kellar and Craft (2020)

## 5 Summary and future directions

The onset and progression of neurodegenerative diseases represent a complex process involving multiple steps and factors. This review emphasizes that environmental pollutants are not merely simple “triggers,” but rather play a crucial role in shaping disease progression by precisely interfering with the core pathogenic processes of pathological proteins. However, mixed exposures, dose-response relationships, the varying long-term impacts of exposures during different life stages, and the existence of genetic susceptibility and gene-environment interactions make predicting the occurrence and course of different diseases exceedingly difficult. Consequently, individuals exhibit diverse phenotypes. Therefore, future efforts should focus on identifying susceptible populations and developing public health prevention strategies for pollution-associated NDs, as well as precise intervention measures for specific exposed groups. First, identifying susceptible populations through large-scale cohort studies integrating exposome data with genetic and epigenetic profiling will be critical for personalized risk prediction. Second, developing public health prevention strategies for pollution-associated neurodegenerative diseases requires stricter regulatory limits on neurotoxic pollutants and promotion of green infrastructure. Third, precise intervention measures for specific exposed groups could include antioxidant supplementation (e.g., N-acetylcysteine, vitamin E) or anti-inflammatory agents targeting microglial activation, though clinical trials are urgently needed. Regarding potential therapeutic targets for environmental factors, emerging strategies deserve exploration. For example, regulating autophagic reaction or proteasome function may counteract pollutant-induced protein aggregation. Besides, modulating the gut-brain axis through probiotics could mitigate peripheral inflammation-driven neurotoxicity. Enhancing blood-brain barrier integrity via tight junction stabilizers might also limit pollutant entry into the brain. These represent promising strategy for pharmacological intervention, though most remain at preclinical stages. Furthermore, advanced multi-omics approaches applied to both human cohorts and animal models will help understand the complex interplay between mixed pollutant exposures and pathological cascades across different life stages more clearly. Finally, longitudinal studies with repeated biomarker measurements in environmentally exposed populations are needed to establish causal links between specific pollutants and the temporal progression of neurodegenerative pathology. By addressing these knowledge gaps, we can move toward a more comprehensive understanding of how environmental factors shape disease trajectories and develop effective strategies to mitigate the growing burden of pollution-related neurological disorders.

## 6 Search strategy and figure preparation

A systematic literature search was performed in PubMed, Embase, and Web of Science to identify studies investigating the relationship between environmental pollutants and the eight hallmark pathogenic proteins of neurodegenerative diseases: amyloid-β, Tau, α-synuclein, TDP-43, FUS, SOD1, PrP, and mHTT. The search strategy combined two concept groups with the operator AND (The Lancet, 2019): environmental pollutants, covering air pollutants (Wimo et al., 2023), metals and heavy metals (Karran and De Strooper, 2022), pesticides and insecticides (de Calignon et al., 2012), industrial chemicals and by-products (Peelaerts et al., 2015), emerging contaminants (Karanth et al., 2020), plastics, and (Nelson et al., 2019) personal care products/endocrine disruptors; and the eight neurodegenerative protein families listed above. Both Medical Subject Headings (MeSH) and free-text keywords were used to maximise sensitivity (the full search string is provided in Table 2). The search was restricted to English-language articles, and the time frame covered recent 50 years.

**TABLE 2:** Search strategy and terms used for systematic literature retrieval.

((“Air Pollution”[MeSH] OR “Particulate Matter”[MeSH] OR pm2.5 OR “air pollutant*” OR “environmental exposure”[MeSH]) OR (“Metals, Heavy”[MeSH] OR “heavy metal*” OR lead OR cadmium OR mercury OR arsenic) OR (“Pesticides”[MeSH] OR pesticide* OR insecticide* OR “Endocrine Disruptors”[MeSH]) OR (“Industrial Waste” OR “industrial chemical*” OR byproduct* OR “emerging contaminant*” OR “plastics” OR “Cosmetics”[MeSH])) AND (“Amyloid beta-Peptides”[MeSH] OR “amyloid beta” OR “amyloid-beta-P“Amyloid“beta-amyloid”) AND (“tau Proteins”[MeSH] OR “tau protein” OR “phospho-tau” OR “p-Tau” OR “Tau”) AND (“alpha-Synuclein”[MeSH] OR ″αlpha-Synuc” OR ″αOR a” OR “alpha-syn” OR “-synuc AND (“Tar DNA-Binding Protein”[MeSH] OR “TDP-43″OR “TARDBP” OR “TDP43″) AND (“FUS Protein, Human”[MeSH] OR “FUS protein” OR “FUS” OR “TLS” OR “TLS/FUS”) AND (“Superoxide Dismutase-1”[MeSH] OR “SOD1″ OR “Cu/Zn-SOD” OR “superoxide dismutase 1″) AND (“Prion Proteins”[MeSH] OR “PrP” OR “PrP^Sc” OR “prion protein” OR “scrapie prion protein”) AND (“Huntingtin Protein”[MeSH] OR “mutant huntingtin” OR “mHTT” OR “polyQ huntingtin” OR “Htt”)

Figures were assembled using icons and schematic assets from BioRender (https://biorender.com) and other publicly available sources like SciDraw, combined with original vector elements. All figures were then arranged, annotated, and finalized in Adobe Illustrator, Microsoft PowerPoint, and Adobe Photoshop.

## Glossary

AD: Alzheimer’s disease
ALS: Amyotrophic lateral sclerosis
APP: Amyloid precursor protein
Aβ: Amyloid-beta
BACE1: Beta-secretase 1
BBB: Blood-brain barrier
BPA: Bisphenol A
CBD: Corticobasal degeneration
CSF: Cerebrospinal fluid
DLB: Dementia with Lewy bodies
DMN: Default mode network
EDC: Endocrine-disrupting chemical
FTD: Frontotemporal dementia
FUS: Fused in sarcoma
GCI: Glial cytoplasmic inclusion
LB: Lewy body
LLPS: Liquid-liquid phase separation
mHTT: Mutant huntingtin
MSA: Multiple system atrophy
ND: Neurodegenerative disease
NFT: Neurofibrillary tangle
NLRP3: NOD-like receptor family pyrin domain containing 3
PAH: Polycyclic aromatic hydrocarbon
PCB: Polychlorinated biphenyl
PD: Parkinson’s disease
PFAS: Per- and polyfluoroalkyl substances
PM2.5: Fine particulate matter (≤2.5 μm)
POP: Persistent organic pollutant
PrP: Prion protein
PSP: Progressive supranuclear palsy
RNS: Reactive nitrogen species
ROS: Reactive oxygen species
SOD1: Superoxide dismutase 1
TDP-43: TAR DNA-binding protein 43
TLR4: Toll-like receptor 4
UFP: Ultrafine particle
UPS: Ubiquitin-proteasome system
α-syn: α-synuclein
